# MediterrAsian Diet Products That Could Raise HDL-Cholesterol: A Systematic Review

**DOI:** 10.1155/2016/2025687

**Published:** 2016-11-01

**Authors:** Mariangela Rondanelli, Attilio Giacosa, Paolo Morazzoni, Davide Guido, Mario Grassi, Gabriella Morandi, Chiara Bologna, Antonella Riva, Pietro Allegrini, Simone Perna

**Affiliations:** ^1^Department of Public Health, Experimental and Forensic Medicine, School of Medicine, Endocrinology and Nutrition Unit, University of Pavia, Azienda di Servizi alla Persona di Pavia, Pavia, Italy; ^2^Department of Gastroenterology, Policlinico di Monza, 20900 Milan, Italy; ^3^Research and Development Department, Indena SpA, 20139 Milan, Italy; ^4^Department of Public Health Experimental and Forensic Medicine, Unit of Biostatistics and Clinical Epidemiology, University of Pavia, Pavia, Italy; ^5^Department of Brain and Behavioral Sciences, Section of Biostatistics, Neurophysiology and Psychiatry, University of Pavia, Pavia, Italy

## Abstract

*Background*. High HDL-cholesterol (HDL-C) values are negatively correlated with cardiovascular diseases. This review analyses the effect of the supplementation with various Mediterranean diet products (artichoke, bergamot, and olive oil) and Asian diet products (red yeast rice) on the HDL-C value in dyslipidemic subjects.* Methods*. A systematic review has been done involving all the English written studies published from the 1st of January 1958 to the 31st of March 2016.* Results*. The results of this systematic review indicate that the dietary supplementation with red yeast rice, bergamot, artichoke, and virgin olive oil has promising effects on the increase of HDL-C serum levels. The artichoke leaf extract and virgin olive oil appear to be particularly interesting, while bergamot extract needs further research and the effect of red yeast rice seems to be limited to patients with previous myocardial infarction.* Conclusions*. Various MediterrAsian diet products or natural extracts may represent a potential intervention treatment to raise HDL-C in dyslipidemic subjects.

## 1. Introduction

Cardiovascular disease (CVD) remains the leading cause of morbidity and mortality worldwide [1]. Hyperlipidemia, resulting from the abnormalities of lipid homeostasis, is a common risk factor for the development of CVD [2]. It is well known that the increase of total cholesterol and particularly of LDL-cholesterol (LDL-C) is positively associated with the risk of CVD, while high values of HDL-cholesterol (HDL-C) are inversely correlated with the risk of CVD [3]. A meta-analysis of four prospective studies (the Framingham Heart Study, the Multiple Risk Factor Intervention Trial (MRFIT), the Lipid Research Clinics Prevalence Mortality Follow-Up Study, and the Coronary Primary Prevention Trial) showed that every 1 mg/dL^−1^ (0.026 mmol/L^−1^) increase in HDL-C is associated with a significant coronary heart disease (CHD) risk reduction of 2% in men and 3% in women [4].

From the pharmacological point of view, statins represent the treatment of choice to reduce the serum levels of total and LDL-cholesterol. However, in both primary and secondary prevention trials with statins, only small, insignificant increases in HDL-cholesterol were achieved. In the Air Force/Texas Coronary Atherosclerosis Prevention Study (AFCAPS/TexCAPS), treatment with lovastatin resulted in a 6% mean increase in HDL-cholesterol [5]; in the West Of Scotland Coronary Prevention Study (WOSCOPS), treatment with pravastatin resulted in a 5% increase [6]; in the Cholesterol and Recurrent Events (CARE) trial, pravastatin also increased HDL-cholesterol by 5% [7]; and in the Scandinavian Simvastatin Survival Study (4S), simvastatin increased HDL-cholesterol by 8% (insignificant when corrected for the placebo group) [8].

Therefore, at the present time, there is no clear indication that HDL-cholesterol elevation by statins translates into clinical benefit [9].

Interesting effects on the serum lipid profile are associated with behavioral interventions. Healthy diet and physical exercise have beneficial effects on the improvement of the serum lipid profile, with reduction of total cholesterol (TC), triglycerides (TG), and LDL-C with increase of HDL-C [10]. A healthy diet may include reducing the intake of saturated and* trans *fatty acids and dietary cholesterol and increasing the intake of fish, fruit, and vegetables. Typically, this objective is achieved with Mediterranean diet, rich in olive oil, fruits and vegetables, whole cereals, nuts, legumes, fish, and red wine [11] and with red yeast rice which is a typical product of the Asian diet [12]. The combined preventive role of these two diet models has been defined as “MediterrAsian diet” [13]. Recent data demonstrate that various MediterrAsian products and botanical extracts such as the extracts of artichoke, bergamot, red yeast rice, or monacolin and olive oil may increase HDL-cholesterol.

Other food or food derived components could be efficacious in favorably modulating human serum HDL-C, such as ethanol and eggs. Prior and recent evidence shows favorable changes in HDL-C, other CVD risk factors, and CVD event rates with moderate and regular ethanol intake [14]. However, the application of these findings in clinical practice remains problematic, due to the lack of randomized, controlled clinical trials and due to the potential hazards of ethanol consumption. As far as eggs are concerned, multiple research data and even meta-analyses have been conducted to investigate the effects of eggs on serum cholesterol level and cardiovascular health with different conclusions [15]. Convincing data have been obtained on the increase of HDL-C after egg consumption in patients with metabolic syndrome [16], but not in healthy subjects.

The purpose of this systematic review is to investigate the effect of the supplementation with the four typical MediterrAsian products previously described (extracts of artichoke and bergamot, red yeast rice or monacolin, and olive oil) on high-density lipoprotein cholesterol (HDL-C).

## 2. Methods

The present systematic review was performed according to the following steps suggested by Egger et al. [17]: (i) configuration of a working group; (ii) formulation of the revision question on the basis of considerations made in the abstract; (iii) identification of relevant studies. The search involved all the studies published from the 1st of January 1958 to the 31st of March 2016. English written articles were identified by searching the Medline database [18], Scopus [19], ISI Web of Science [20], and Google Scholar [21]. The analysis was carried out in the form of a systematic review of the reports.

### 2.1. Inclusion and Exclusion Criteria

Two reviewers (Simone Perna and Mariangela Rondanelli) independently reviewed each report. For each of the relevant abstracts, full publications were retrieved for evaluation on the basis of criteria established a priori. Original clinical trials investigating the effects of botanical extract supplementations as red yeast rice, monacolin k, bergamot, artichoke, and olive oil and their eventual comparison with a control diet (if available) were evaluated. The change of high-density lipoprotein cholesterol (HDL-C) was the primary outcome and no secondary outcomes were considered.

**Figure 1:**
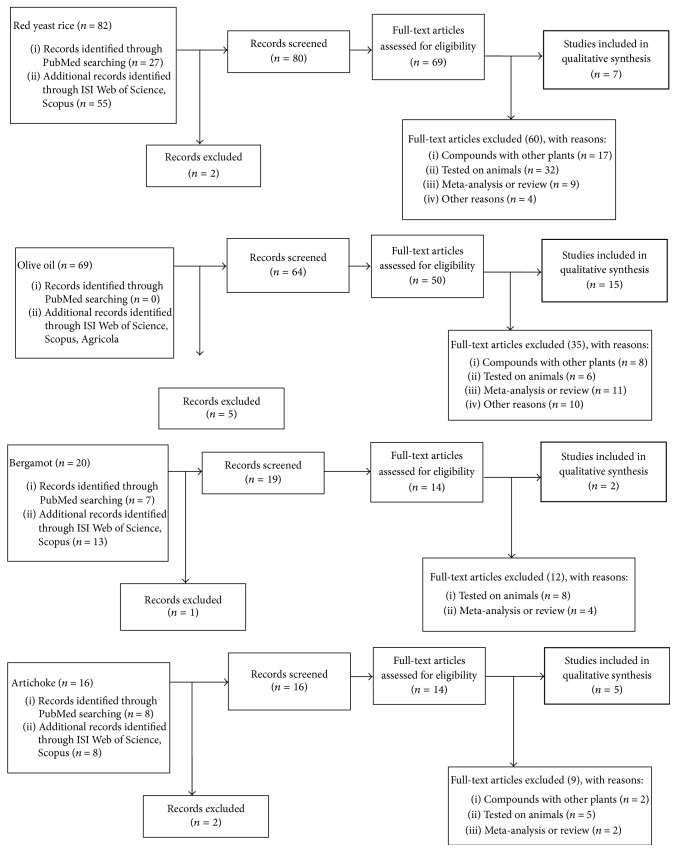
Flow diagram of the study divided by botanical supplementation.

All adult age groups were included. The eligible studies were required to report baseline and follow-up values, that is, the mean change from baseline (Δ-change) and/or the mean difference Δ-changes (MDΔ) between intervention groups for HDL-C outcome. Trials were included also if a botanical extract supplementation was associated with the supplementation of other foods.  Figure 1 reports the flow diagram of the study divided by botanical supplementation.

### 2.2. Data Collection

The following data were extrapolated from all the revised studies: (i) author and year of publication; (ii) number of participants for each study; (iii) mean age of the subjects; (iv) inclusion criteria; (v) dietary supplement (in intervention and control groups); (vi) duration of intervention (in weeks); (vii) mean baseline HDL-C; (viii) mean postintervention HDL-C; (ix) mean change from baseline (Δ-change); (x) mean difference Δ-change (MDΔ) between intervention groups for HDL-C outcome (*P* values); (xi) study design and level of evidence (as suggested by Centre for Evidence-Based Medicine [29]).

The obtained data were summarized in four tables (Tables 1 [Table tab2] [Table tab3]–4). Each table shows study design, level of evidence, inclusion criteria, sample size, baseline mean age, dietary intervention∖control, and duration. HDL-C related measurements, that is, Δ-changes and/or MDΔ, if available, were included. In order to define the type of intervention, we focused on the dosage of supplementation (grams per day), on the number of supplementation days, on the number of patients (control and∖or intervention group), and on the type of control diet.

## 3. Results

### 3.1. Red Yeast Rice and Monacolin K

Concerning the effects of red yeast rice on HDL-C, the literature search based on the keywords [“Monacolin k” OR “Monascus Purpureus” OR “Red Yeast Rice”] AND [“HDL” OR “cholesterol” OR “high density lipoproteins”] retrieved 82 articles. After screening, 69 papers were selected for full-text revision. After applying our inclusion and exclusion criteria, 62 studies were excluded and 7 clinical trials were selected for the present systematic review.  Figure 1 shows the study selection process. The 7 clinical trials included a total of 5444 adults (4224 females, 1620 males).

The trial characteristics are outlined in Table 1. Regarding the study design, out of the 7 selected studies, two were randomized, double-blind, placebo-controlled, parallel-groups trials [22, 28], two were randomized, double-blind, and placebo-controlled trials [25, 27], one was a randomized and double-blind trial [24], one was a randomized and open label parallel trial [26], and one was a pre-post study [23].

Concerning the control group, four studies [22, 25, 27, 28] were placebo-controlled, one study [24] was performed versus pravastatin 40 mg/day, and one [26] was performed versus a hypocholesterolemic diet. The study of Lee et al. [23] had no control group.

The baseline characteristics and disease status of the participants varied. Four studies [22, 23, 25, 27] included subjects with hypercholesterolemia; two studies [26, 28] included subjects with moderate hypercholesterolemia or normal lipid levels. The remaining study [24] included subjects (i) with statin or red yeast rice treatments during the month before randomization, (ii) with a history of statin-associated myositis or rhabdomyolysis, and (iii) with a history of generalized chronic pain.

Dietary interventions lasted from 8 to 216 weeks, with a dosage of red rice yeast ranging from 1200 to 4800 mg/die or a dosage of Xuezhikang (XZK) ranging from 1200 to 2400 mg/die or a dosage of* Monascus purpureus* of 200 mg/die.

The study by Lu et al. [28] based on a large number of cases (4870) showed a statistically significant increase of HDL-C (Δ% = +4.2%, *P* < 0.001) after supplementation with XZK versus placebo, while the other six studies did not show significant changes of HDL-C. The randomized, placebo-controlled, multicentre study coordinated by Lu et al. was conducted to evaluate the effects of XZK, a partially purified extract of red yeast rice, on the lipid pattern and on various cardiovascular end points in Chinese patients with a previous myocardial infarction. This study differs from all the other research projects because it is based on a very large number of patients with previous myocardial infarction (nearly 5000) who were treated for long time (4.5 years). Beside showing a significant increase of HDL-C as well as a significant decrease of TC, LDL-C, and TG, this study showed relevant clinical results. The frequency of nonfatal myocardial infarction and death from coronary heart disease showed an absolute and relative decrease of 4.7% and 45%, respectively. Treatment with XZK also significantly decreased cardiovascular and total mortality by 30% and 33% and the need for coronary revascularization by 1/3.

In conclusion, the effect of red yeast rice on HDL-C is still a matter of debate, because the only study that showed positive results follows a research protocol which differs from all the other studies. In this project, the selected population was constituted by patients with previous myocardial infarction. The strength of the study is that it has been conducted for a very long time and with recruitment of a very large population sample. The analysis of the data of Lu et al. shows that the effects of XZK on HDL-C may be caused at least in part by the potential properties of its nonstatin components. Thus, it is likely that components other than lovastatin in red yeast rice, such as lovastatin hydroxy acid, plant sterols, isoflavones, and isoflavone glycosides, may contribute to the results [28]. However, the future use of this product in clinical practice will depend on the separation, identification, characterization, and development of a carefully formulated preparation of red yeast rice as well as on additional studies that confirm its effect on a favorable lipid pattern in patients with dyslipidemia without previous myocardial infarction.

### 3.2. Olive Oil

Concerning the effects of olive oil on HDL-C, the literature search based on the keywords [“Olive Oil” OR “EVO” OR “Virgin Olive Oil”] AND [“HDL” OR “cholesterol” OR “high density lipoproteins”] retrieved 69 articles. After screening, 50 papers were selected for full-text revision. After applying our inclusion and exclusion criteria, 35 studies were excluded and 15 clinical trials were selected for the present systematic review.  Figure 1 shows the study selection process. The 15 clinical trials studied a total of 1053 adults.

The trial characteristics are shown in Table 2. Regarding the study design, out of the 15 studies, two were randomized, double-blind, placebo-controlled, and parallel-groups trials [32, 42], two were randomized, double-blind, and controlled clinical trials [30, 35], one was a randomized and double-blind trial [39], one was a randomized, controlled, and parallel study [44], one was a randomized, double-blind, and controlled crossover feeding trial [31], one was a randomized and single-blind crossover trial [33], two were randomized and controlled crossover studies [34, 38], one was a randomized and intervention crossover trial [36], two were double-blind crossover studies [37, 43], and two were pre-post studies [40, 41].

Regarding the control group, two studies used DHA [30, 32], one study used corn oil [31], two studies used low polyphenol olive oil [34, 36], one used virgin argan oil [44], and one used fish oil [30]. One study [33] had three control groups (Group A: EPA: 3 g/day; Group B: DHA: 3 g/day; Group C: fish oil: 3 g/day). One study [35] had two control groups (Group A: conjugated linoleic acid, free fatty acids: 27 g/day; Group B: conjugated linoleic acid, triacylglycerol: 27 g/day). Another study [37] had two control groups (Group A: 75 g/day of refined olive oil (OO); Group B: 75 g/day of common olive oil (a mixture of refined and virgin olive oil)).

One study had a control group which maintained its own dietary habits [39]. Four studies had no control group [38, 40, 41, 43].

The baseline characteristics and disease status of the participants varied. Four studies [30, 33, 36, 42] included subjects with hypercholesterolemia or moderate hypercholesterolemia; seven studies [37–41, 43, 44] included subjects with normal lipid levels, one study [32] included subject with adherence to a vegetarian diet for at least one year, and two studies [34, 35] included healthy males and healthy volunteers.

Dietary interventions lasted from 2 to 52 weeks with a dosage of extra virgin olive oil ranging from 2.28 to 54 g/day or a dosage of high polyphenol olive oil (HPOO) ranging from 22 to 69 g/day or with a dosage of virgin oil of 75 g/day. Two studies [38, 43] had three intervention groups (first study: Group A: HPOO (high polyphenol olive oil): 22 g/day; Group B: MPOO (medium polyphenol olive oil): 22 g/day; Group C: LPOO (low polyphenol olive oil): 22 g/day; second study: Group A: HPOO: 25 g/day; Group B: MPOO: 25 g/day; Group C: LPOO: 25 g/day).

Two studies [32, 38] showed a statistical significant change (*P* < 0.05) in HDL-C level between intervention and control groups. These three studies included a high number of participants; in particular, the study performed by Covas et al. in 2006 enrolled 200 subjects. Three studies [37, 39, 40] showed a significant difference (Δ-changes = 0.08; 0.17; 0.16 mmol/L, *P* < 0.05) between pre- and posttreatment only in the intervention groups. These studies enrolled a small sample of participants (<100 subjects). One study [44] showed a significant change in both the intervention group (*P* < 0.05) and the control group (*P* < 0.05). One crossover study [43] showed a significant difference in HDL-C levels in two out of the three intervention groups (*P* < 0.05 in Group A and Group B). In the other three studies [30, 31, 36], no significant changes were observed, but it has to be considered that the selected population was characterized by normal or slightly increased TC values (5,81, 5.32, and 4.19 mmol/L) and with an HDL, respectively, equal to 1.21, 1.09, and 1.52 mmol/L. In addition, these studies included a small sample of participants (57, 54, and 46 subjects).

In conclusion, these data provide evidence that polyphenol-rich olive oil favors the enhancement of HDL-C. The health-promoting properties of olive oil have traditionally been attributed to the oleic acid (omega-9 monounsaturated fatty acid, 18:1 n-9) component of olive oil. As a matter of fact, oleic acid enhances the levels of high-density lipoprotein (HDL) and produces a small drop in low-density lipoprotein [52, 53]. However, current knowledge indicates that olive oil polyphenols such as hydroxytyrosol play an important role in fortifying the healthy effects of olive oil [54]. As a phenolic acid, hydroxytyrosol decreases TC, LDL-C, and TG, while it increases HDL-C in hyperlipidemic rabbits and rats [55–58]. The purified polyphenols clinical studies are scarce and responsive mechanisms are not yet distinct. Olive oil polyphenols promote the main HDL antiatherogenic function, that is, its cholesterol efflux capacity. These polyphenols increase HDL size, promote a greater HDL stability reflected as a triglyceride-poor core, and enhance the HDL oxidative status, through an increase in the olive oil polyphenol metabolites content in the lipoprotein [34]. Covas et al. in 2006 with a 15-week-supplementation of 2.2 g of the polyphenol content of olive oil showed further benefits on HDL-cholesterol levels and oxidative damage in addition to those obtained with its monounsaturated fatty acid content. This study provides additional evidence to recommend the use of polyphenol-rich olive oil, that is, virgin olive oil, as a source of fat capable of promoting a reduction of some cardiovascular risk factors including the increase of HDL-C [38].

### 3.3. Bergamot

Concerning the effects of bergamot extract (BPF) on serum HDL-C, the literature search based on the keywords “bergamot” and [“HDL” OR “cholesterol” OR “high density lipoproteins”] retrieved 20 articles. After screening, 14 papers were selected for full-text revision. After applying our inclusion and exclusion criteria, 12 studies were excluded and 2 clinical trials were selected for the present systematic review.  Figure 1 shows the study selection process. The 2 clinical trials studied a total of 314 adults (gender characteristics were not specified). The trial characteristics are outlined in Table 3. Regarding the study design, one study was a randomized double-blind placebo-controlled trial [56], while the other one was an open label controlled trial [45].

Concerning the control group, Mollace et al.'s study [46] was placebo-controlled, whereas in Gliozzi et al.'s study [45] there were 4 control groups (Group A: rosuvastatin 10 mg/day; group B: rosuvastatin 20 mg/day; group C: BPF 1000 mg + rosuvastatin 10 mg/day; and group D: placebo).

The baseline characteristics and disease status of the participants varied. Gliozzi et al.'s study [45] included subjects with mixed hypercholesterolemia, whereas in the other study 4 different groups were included: (i) subjects with hypercholesterolemia, (ii) hyperlipidemia (hypercholesterolemia and hypertriglyceridemia), (iii) hyperlipidemia and glycemia over 110 mg/dL, and (iv) patients who discontinued statin therapy for muscular pain and a significant elevation of serum creatine phosphokinase (CPK). Dietary interventions lasted 4 weeks with a dosage of bergamot juice ranging from 500 to 1000 mg/die.

One study [45] with a small sample of participants (77 subjects) showed a statistically significant increase (*P* < 0.05) of the HDL-C value. Similar results were obtained in the Mollace et al. study [46].

Similar results were obtained in study performed by Mollace et al. [46]. In this study a group of 59 patients diagnosed with metabolic syndrome were supplemented with BPF treatment with 500 mg/day (group C1, *n* = 20) or 1000 mg/day (group C2, *n* = 19) or placebo (group CPL, *n* = 20). The metabolic syndrome patients, suffering from HC/HT/HG, responded very well to BPF therapy. After the treatment with high BPF dose, the patients with metabolic syndrome presented a significant mean increase (+29.6%, *P* < 0.01) in HDL-C only in the 1000 mg BPF treatment group (C2 group).

In conclusion, exogenous supply of bergamot extract which is rich in natural polyphenols favorably modulates various peripheral biomarkers of cardiometabolic risk, including the increase of HDL-cholesterol. In addition, a reduction of serum fasting glycaemia was observed. As a matter of fact, BPF inhibits HMG-CoA reductase activity and enhances reactive vasodilation, thus representing an efficient phytotherapeutic approach to fight glycemic and lipidemic disorders. A major contribution to the serum lipemic response observed in patients undergoing BPF treatment seems to be the modulatory properties of the flavonone glycoside components of the bergamot juice extract: in particular, naringin and neohesperidin seem to be of relevant interest.

Moreover, bergamot-derived polyphenols enhance the effect of rosuvastatin in normalizing the serum lipemic profile, allowing a potential reduction in daily rosuvastatin doses. The small number of studied patients as well as the availability of only two clinical studies coming from a unique research group suggests the need of additional research projects on this very interesting natural extract, in order to confirm the available data.

### 3.4. Artichoke

Concerning the effects of artichoke leaf extract (ALE) on HDL-C, the literature search based on the keywords [“Artichoke” OR “Artichoke Leaf Extract”] and [“HDL” OR “cholesterol” OR “high density lipoproteins”] retrieved 16 articles. After screening, 14 papers were selected for full-text revision. After applying our inclusion and exclusion criteria, 9 studies were excluded and 5 clinical trials were selected for the present systematic review.  Figure 1 shows the study selection process. The 5 clinical trials studied a total of 395 adults.

The trial characteristics are outlined in Table 4. Regarding the study design, out of the 5 studies, three were randomized, double-blind, and placebo-controlled trials [47, 48, 50], one was a randomized, double-blind, and placebo-controlled multicentre clinical trial [51], and one was a placebo-controlled trial [49]. The baseline characteristics and disease status of the participants varied. Two studies included subjects with hypercholesterolemia (Englisch et al. [51] TC: 7.7 mmol/L; Bundy et al. [48] TC: 7.16 mmol/L); one study included subjects with moderate hypercholesterolemia (mean value: 6.5 mmol/L) [50]. The remaining two studies included subjects with type 2 diabetes without insulin therapy and with moderate hypercholesterolemia (mean values: 5.9 and 6.1 mmol/L, resp.). In one of these two studies, the mean basal HDL-C value was low (0.88 mmol/L) [49], while it was high in the other one (1.46 mmol/L) [47].

Dietary interventions lasted from 6 to 12 weeks (mean value: 64 days) with a supplementation of artichoke leaf extract (dosage from 0.5 g/day to 6 g/day), dry extract (dosage of 1.8 g/day), and flowering bud leaf extract (dosage of 0.6 g/day).

One study [50] showed a statistically significant difference (Δ-changes = +0.20 mmol/L, *P* < 0.01, 95% CI = 0.07; 0.34) in the level of HDL-C when intervention and control group were compared. Similar results were obtained in Nazni et al. study [49] when pre-post intervention data were compared (Δ-change = +0.22 mmol/L). In the other three studies [47, 48, 51], no significant HDL-C changes were observed. Anyhow, looking carefully at the mean basal lipemic values of the selected samples of all the five studies, a significant increase of HDL-C was not obtained when the mean TC value was higher than 7.16 or when mean HDL-C was higher than 1.46 mmol/L and the three negative studies showed basal lipemic values above these values.

In conclusion, ALE supplementation appears a useful treatment to increase HDL-C provided that basal TC or basal HDL-C is not too high. Therefore, ALE could represent a potential treatment option for subjects with primary mild hypercholesterolaemia, particularly if HDL values are low. ALE also proved to be safe and well tolerated. Future studies are needed to confirm the favorable clinical results and safety over a longer duration of intervention with ALE.

Although the mechanisms explaining the HDL-cholesterol increasing effect of ALE are not well known, the most likely explanation could be related to their polyphenolic content and in particular to chlorogenic acid [59–61]. This compound could favor the increase in HDL-C through the enhancement of the activity of paraoxonase-1 (PON-1). PON-1 is an enzyme associated with HDL which prevents the oxidation of HDL-C, thus favoring its antioxidative and anti-inflammatory effects [62]. Gugliucci and Bastos demonstrated that chlorogenic acid protects PON-1 activity in HDL-C by means of an in vitro study. In this experiment, chlorogenic acid protects PON-1 activity in human HDLs from inactivation caused by hypochlorite (HOCl), at concentrations of HOCl (50 *μ*M) and chlorogenic acid (2–10 *μ*M) compatible with those found in humans [63].

Previous human studies proved that the increase in PON-1 activity correlates strongly with the increase in HDL-C [64, 65]. Based on these data, it can be hypothesized that chlorogenic acid, present in ALE extract, may induce an increase in PON-1 activity and, as a consequence, may favor the increase in HDL-C in humans.

## 4. Conclusions

**Figure 2:**
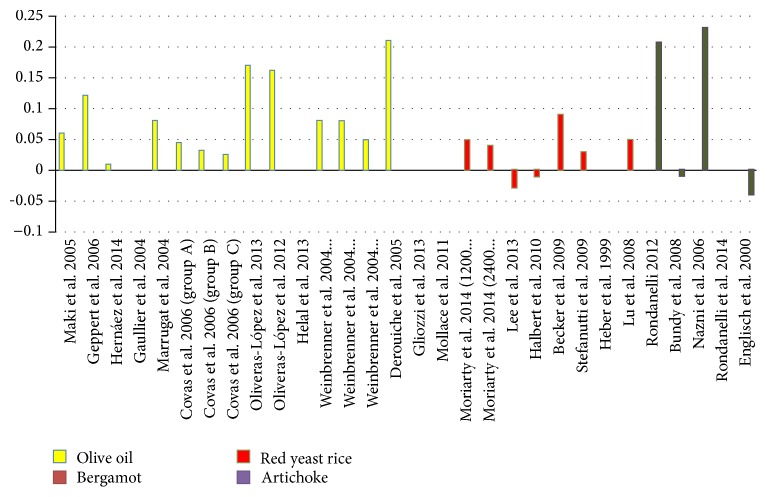
Variation of HDL-C in mmol after intervention.

The results of this systematic review indicate that the dietary supplementation with four typical components of the MediterrAsian diet, that is, red yeast rice, bergamot, artichoke, and virgin olive oil, has promising effects on the increase of HDL-cholesterol serum levels. In Figure 2, variation of HDL-C in mmol after intervention has been shown. The artichoke leaf extract and virgin olive oil appear to be particularly interesting: for both these products, a good number of papers are available as well as a large number of studied subjects. Moreover, the entity of the HDL-C increase appears to be relevant after supplementation with both the artichoke leaf extract and the virgin olive oil, while this result does not appear with “nonvirgin” olive oil. Anyhow, a new meta-analysis of the existing data should be planned for both of them, in order to obtain a more accurate and evidence-based information. Additional research should be planned on bergamot extract because the available results on this botanical appear very interesting but based on a too small number of studies (two papers coming from a unique research group) and of evaluated subjects. The effect of red yeast rice on HDL-C appears positive only after long term intervention in patients with previous myocardial infarction; while negative effects are shown in multiple studies conducted in asymptomatic dyslipidemic subjects. This finding could play a role in the treatment of patients with positive history of myocardial infarction, but this finding needs further confirmation by means of additional controlled intervention studies. The effects of these MediterrAsian natural extracts and food products on HDL-C changes appear very promising, but further research by means of additional well conducted, randomized, controlled studies in a large male and female population with low HDL-C values is needed. Moreover, the effect on both HDL-cholesterol and HDL particles should be evaluated, as well as the correlation between the increase in HDL-cholesterol and nonfunctional HDL particles.

## Figures and Tables

**Table 1:** Red rice yeast.

First author, year [ref]	Number of participants (M-F)	Age, y	Inclusion criteria	Dietary supplement		Duration (wk)	Mean or median baseline HDL-C (mmol/L)	Mean post-HDL-C (mmol/L)	Δ change (within groups) (*P* valve)	MDΔ (between groups) (*P* value)	Study design (level of evidence)^*∗*^
				Intervention	Control						
Moriarty, 2014 [22]	116 (30-86)	56.70 ± 10.80	Aged ≥ 18 years with TC ≥ 6.2 mmol/L, LDL-C ≥ 4.14 mmol/L (but ≤5.69 mmol/L), and TG < 4.52 mmol/L. Other requirements included a BMI < 36 kg/m^2^.	Group A: XZK: 1200 mg/die *Patients: 36* Group B: XZK: 2400 mg/die *Patients: 29*	Placebo *Patients: 38*	12	I: Group A: 1.57 ± 0.33 Group B: 1.58 ± 0.48 C: 1.48 ± 0.31	I: Group A: 1.62 ± 0.35 Group B: 1.62 ± 0.45 C: 1.45 ± 0.39	I: Group A: +0.05 (*P* = NS) Group B: +0.04 (*P* = NS) C: −0.03 (*P* = NS)	I(A)-C: +0.08 (*P* = NR) I(B)-C: +0.07 (*P* = NR)	Randomized, double-blind, placebo-controlled, parallel-groups trial (Level 2)

Lee, 2013 [23]	30 (19-11)	45.60 ± 12.60	Aged > 18 years, LDL-C > 4.14 mmol/L, or TG > 2.26.	RRY: 1200 mg/die *Patients: 30*	None	8	1.05 (0.23)^∧^	NR	−0.03 (*P* = NS)	NE	Pre-post study (Level 3)

Halbert, 2010 [24]	43 (11-32)	62.65 ± 7.75	Statin or red yeast rice use during the month before randomization, a history of statin-associated myositis or rhabdomyolysis, and a history of generalized chronic pain.	RRY: 4800 mg/die *Patients: 17*	Pravastatin: 40 mg *Patients: 22*	12	I: 1.32 ± 0.42 C: 1.37 ± 0.43	I: 1.31 ± 0.38 C: 1.37 ± 0.44	I: −0.01 (*P* = NR) C: 0 (*P* = NR)	−0.01 (*P* = NR)	Randomized, double-blind trial (Level 2)

Becker, 2009 [25]	62 (22-40)	61.00 ± 8.75	Age from 21 to 80 years with hypercholesterolemia. LDL-C > 2.6 mmol/L or <5.5 mmol/L, TG ≥ 4.4 mmol/L.	RRY: 1800 mg/die *Patients: 30*	Placebo *Patients: 29*	24	I: 1.37 ± 0.31 C: 1.33 ± 0.36	I: 1.46 ± 0.32 C: 1.40 ± 0.38	I: 0.09 (*P* = NS) C: 0.07 (*P* = NS)	0.02 (*P* = NR)	Randomized, double-blinded, placebo-controlled trial (Level 2)

Stefanutti, 2009 [26]	240 (110-130)	56.50 ± 9.00	Primary moderate hypercholesterolemia. No cardiovascular disease, hypertension, diabetes, or obesity.	MP: 200 mg/die *Patients: 110*	Hypocholesterolemic diet *Patients: 130*	32	I: 1.37 ± 0.34 C: 1.40 ± 0.36	I: 1.40 ± 0.39 C: 1.32 ± 0.47	I: 0.03 (*P* = NS) C: −0.08 (*P* = NS)	0.10 (*P* = ns)	Randomized, open-labeled, parallel-groups trial (Level 2)

Heber, 1999 [27]	83 (46-37)	—	LDL-C > 4.14 mmol/L, TG < 2.94 mmol/L, and having not been treated previously for hypercholesterolemia.	RRY: 2400 mg/die *Patients: 42*	Placebo *Patients: 41*	12	I: 1.29 ± 0.34 C: 1.19 ± 0.26	I: 1.29 ± 0.36 C: 1.19 ± 0.28	I: 0.00 (*P* = NS) C: 0.00 (*P* = NS)	0 (*P* = NR)	Randomized, double-blind, placebo-controlled prospective study (Level 2)

Lu, 2008 [28]	4870 (3986-884)	60.40 ± 8.43	No cardiovascular disease, diabetes, renal or hepatic disease, systolic blood pressure < 180 mmHg or diastolic blood pressure < 110 mmHg, and fasting plasma glucose < 200 mg/dL. TC from 4.4 to 6.46 mmol/L, TG ≤ 4.52 mmol/L. LDL-C > 4.65 mmol/L at screening could be retested after 4 weeks of dietary therapy.	XZK: 1200 mg/die *Patients: 2429*	Placebo *Patients: 2441*	182	I: 1.19 ± 0.39 C: 1.19 ± 0.36	I: 1.24 ± 0.31 C: 1.19 ± 0.31	I: 0.05 (*P* = NS) C: 0.00 (*P* = NS)	0.05 (*P* < 0.001)	Randomized, double-blind, placebo-controlled, parallel-groups study (Level 2)

MDΔ: mean difference Δ change.

I: intervention; C: control.

*P*: *P* value; NS: not significant (*P* > 0.05); NR: not recorded; NE: not expected.

XZK: Xuezhikang.

MP: *Monascus purpureus*.

RRY: red rice yeast.

^*∗*^As suggested by Centre for Evidence-Based Medicine [29].

^∧^Median (IQR).

**Table 2 tab2:** Olive oil.

First author, year [ref]	Number of participants (M-F)	Age, y	Inclusion criteria	Dietary supplement		Duration (wk)	Mean or median baseline HDL-C (mmol/L)	Mean post-HDL-C (mmol/L)	Δ change (within groups) (*P* value)	MDΔ (between groups) (*P* value)	Study design (level of evidence)^*∗*^
				Intervention	Control						
Maki, 2015 [30]	57 (31-26)	53.6 ± 2.5	HDL-C ≤ 1.14 mmol/L for men and ≤1.40 mmol/L for women but ≥0.91 mmol/L. BMI < 40 Kg/m^2^. TC < 7.76 mmol/L or TG < 3.95 mmol/L. Systolic blood pressure < 160 mmHg and/or diastolic blood pressure < 100 mmHg.	EVO: 2.6 g/die *Patients: 30*	DHA: 1.52 g/die *Patients: 27*	6	I: 1.10 ± 0.06 C: 1.09 ± 0.10	NR	I: +0.06 (*P* = NR) C: +0.1 (*P* = NR)	−0.04 (*P* = NS)	Randomized, double-blind, controlled clinical trial (Level 2)

Maki, 2005 [31]	54 (19-35)	53.8 ± 1.3	Age from 18 to 74 years. BMI ≥ 18.5 Kg/m^2^ and <35 Kg/m^2^. LDL-C > 3.36 mmol/L and <5.17 mmol/L. TG ≤ 3.95 mmol/L. Energy needs of ≥1800 Kcal/d.	EVO: 54 g/die *Patients: 27*	Corn Oil: 54 g/die *Patients: 27*	9 (6 + 3 washout)	1.23 ± 0.04	I: 1.20 ± 0.04 C: 1.18 ± 0.04	NR	NR	Randomized, double-blind, controlled crossover feeding trial (Level 2)

Geppert, 2006 [32]	106 (25-81)	25.9 ± 5.6	Adherence to a vegetarian diet for at least one year. Age ≥ 18 years. BMI between 18 and 25 Kg/m^2^.	EVO: 2.28 g/die *Patients: 53*	DHA-rich oil from microalgae: 2.28 g/die *Patients: 53*	8	I: 1.65 ± 0.06 C: 1.67 ± 0.06	I: 1.77 ± 0.06 C: 1.66 ± 0.06	I: +0.12 (*P* = 0.002) C: −0.1 (*P* = NS)	0.13 (*P* = 0.002)	Randomized, double-blind, placebo-controlled, parallel-groups (Level 2)

Rambjør, 1996 [33]	69 (25-44)	Group A: 30.1 ± 6.6 Group B: 31.1 ± 11.8 Group C: 28.9 ± 7.6	Normolipidemia except TG levels in the higher end of normal (50th–90th percentiles for their age and sex).	Group A: EVO: 5 g/die *Patients: 25* Group B: EVO: 5 g/die *Patients: 9* Group C: EVO: 5 g/die *Patients: 35*	Group A: EPA: 3 g/die *Patients: 25* Group B: DHA: 3 g/die *Patients: 9* Group C: fish oil: 3 g/die *Patients: 35*	5 (3 + 2 washout)	C: Group A: 1.21 ± 0.32 Group B: 1.25 ± 0.31 Group C: 1.27 ± 0.32	Group A: I: 1.17 ± 0.31 C: 1.24 ± 0.34 Group B: I: 1.18 ± 0.31 C: 1.20 ± 0.35 Group C: I: 1.34 ± 0.34 C: 1.38 ± 0.38	Group A: I: NR (*P* = NR) C: 0.03 (*P* = NR) Group B: I: NR (*P* = NR) C: −0.01 (*P* = NR) Group C: I: NR (*P* = NR) C: +0.11 (*P* = NR)	Group A: I-C: NR (*P* = NS) Group B: I-C: NR (*P* = NS) Group C: I-C NR (*P* = NS)	Randomized, single-blind, crossover study (Level 2)

Hernáez, 2014 [34]	47 (47-0)	33.5 ± 10.9	Healthy males	HPOO: 25 g/die *Patients: 47*	LPOO: 25 g/die *Patients: 47*	10	I: 1.41 ± 0.31 C: 1.37 ± 0.30	I: 1.40 ± 0.33 C: 1.40 ± 0.30	I: −0.01 (*P* = NS) C: 0.03 (*P* = NS)	−0.04 (*P* = NS)	Randomized, crossover, controlled study (Level 2)

Gaullier, 2004 [35]	180 (31-149)	I: 45 ± 9.5 C: Group A: 44.5 ± 10.7 Group B: 48 ± 10.7	Healthy volunteers aged 18–65 years and BMI of 25–30 Kg/m^2^.	EVO: 27 g/die *Patients: 59 *	Group A: Conjugated linoleic acid – free fatty acids: 27 g/die *Patients: 61* Group B: conjugated linoleic acid – triacylglycerol: 27 g/die *Patients: 60*	52	I: 1.50 ± 0.38 C: Group A: 1.40 ± 0.32 Group B: 1.50 ± 0.34	I: 1.50 ± 0.45 C Group A: 1.40 ± 0.38 Group B: 1.40 ± 0.33	I: 0.00 (*P* = NS) C: Group A: −0.03 (*P* = NS) Group B: −0.09 (*P* < 0.05)	I-C(A): +0.03 (*P* = NS) I-C(B): +0.09 (*P* = NS)	Randomized, double-blind, placebo-controlled study (Level 2)

Vissers, 2001 [36]	46 (15-31)	38	Age > 17 years. TC < 7 mmol/L. TG < 2.3 mmol/L.	HPOO: 69 g/die *Patients: 46*	LPCOO: 69 g/die Patients: 46	8 (6 + 2 washout)	NR	I: 1.52 ± 0.37 C: 1.54 ± 0.36	NR	−0.01 (*P* = NS)	Randomized, crossover, intervention trial (Level 2)

Marrugat, 2004 [37]	30 (NR-NR)	NR	BMI < 30 kg/m^2^. No dyslipidemia, diabetes, and celiac or other intestinal diseases.	Virgin OO: 75 g/die *Patients: 30*	Group A: 75 g/die refined OO Patients: 30 Group B: 75 g/die common OO Patients: 30	11 (9 + 2 washout)	I: 1.57 ± 0.29 C: Group A: 1.58 ± 0.34 Group B: 1.57 ± 0.34	I: 1.65 ± 0.32 C: Group A: 1.62 ± 0.34 Group B: 1.56 ± 0.31	I: 0.08 (*P* = 0.029) C: Group A: 0.04 (*P* = NS) Group B: −0.01 (*P* = NS)	I-C(A): +0.04 (*P* = NR) I-C(B): +0.09 (*P* = NR)	Placebo-controlled, crossover, double-blind study (Level 3)

Covas, 2006 [38]	200 (NR-NR)	NR	Age range of 20–60. No hyperlipidemia, obesity, diabetes, hypertension, and intestinal disease.	I: Group A: HPOO: 22 g/die *Patients: 183* Group B: MPOO: 22 g/die Patients: 184 Group C: LPOO: 22 g/die *Patients: 182*	None	15 (9 + 6 washout)	NR	Group A: 1.30 (SD NR) Group B: 1.27 (SD NR) Group C: 1.27 (SD NR)	Group A: +0.045 (*P* < 0.05) Group B: +0.032 (*P* < 0.05) Group C: +0.025 (*P* < 0.05)	A-B: 0.025 (*P* < 0.05) A-C: +0.029 (*P* < 0.05) B-C: +0.006 (*P* = NS)	Randomized, crossover, controlled trial (Level 2)

Oliveras-López, 2013 [39]	62 (15-47)	81.7 ± 6.3	65–96 years of age range. No hypertension, diabetes, hyperlipidemia, renal disease, and cardiovascular disease.	EVO: 50 g/die *Patients: 39*	Maintaining dietary habits *Patients: 23*	6	I: 1.42 ± 0.21 C: 1.39 ± 0.24	I: 1.59 ± 0.15 C: 1.41 ± 0.23	I: 0.17 (*P* = 0.005) C: 0.02 (*P* = NS)	0.15 (*P* = NR)	Randomized, double-blind trial (Level 2)

Oliveras-López, 2012 [40]	20 (8-12)	26 ± 2	Volunteers 20–30 years old. BMI between 18 and 25 kg/m^2^. Normolipidemic, no hypertension, diabetes, and cardiovascular disease.	EVO: 50 g/die *Patients: 20*	None	6	1.78 ± 0.30	1.94 ± 0.13	0.16 (*P* = 0.024)	*NE*	Pre-post study (Level 3)

Helal, 2013 [41]	26 (8-18)	20.7 ± 53.3	Age between 25 and 83 years, normal serum lipid profile and blood pressure.	Raw EVO: 25 g/die *Patients: 26*	None	12	1.5 ± 0.07	1.5 ± 0.06	0 (*P* = NS)	*NE*	Pre-post study (Level 3)

Lovegrove, 2004 [42]	84 (NR-NR)	47.8 ± 11.8	Age between 25 and 70 years. BMI between 20 and 37 kg/m^2^. TC < 8 mmol/L, TG < 4 mmol/L. No hypertension, diabetes, and cardiovascular disease.	EVO: 4 g/die	Fish oil: 4 g/die	12	I: 1.4 ± 0.4 C: 1.35 ± 0.35	NR	NR	−0.03 (*P* < 0.05)	Randomized, double-blind, placebo-controlled, parallel study (Level 2)

Weinbrenner, 2004 [43]	12 (12-0)	21.1	Healthy men. BMI < 30 kg/m^2^. No diabetes, hyperlipidemia, and intestinal disease.	I: Group A: HPOO: 25 g/die *Patients: 183* Group B: MPOO: 25 g/die *Patients: 184* Group C: LPOO: 25 g/die *Patients: 182*	None	2	Group A: 1.2 ± 0.08 Group B: 1.16 ± 0.09 Group C: 1.2 ± 0.08	Group A: 1.28 ± 0.08 Group B: 1.24 ± 0.10 Group C: 1.25 ± 0.09	Group A: +0.08 (*P* < 0.05) Group B: +0.08 (*P* < 0.05) Group C: +0.05 (*P* = NS)	A-B: 0 (*P* = NR) A-C: +0.03 (*P* = NR) B-C: +0.03 (*P* = NR)	Randomized, double-blind, crossover study (Level 2)

Derouiche, 2005 [44]	60 (60-0)	23.4 ± 3.8	Male aged between 20 and 43 years with normal BMI. No hypertension, diabetes, hypercholesterolemia, and hypertriglyceridemia.	EVO: 25 g/die *Patients: 30*	Virgin argan oil: 25 g/die *Patients: 30*	3	I: 1.06 ± 0.21 C: 1.17 ± 0.17	I: 1.27 ± 0.27 C: 1.28 ± 0.18	I: 0.21 (*P* = 0.001) C: 0.11 (*P* = 0.012)	0.10 (*P* = NR)	Randomized, controlled, parallel study (Level 2)

MDΔ: mean difference Δ change.

I: intervention; C: control.

*P*: *P* value; NS: not significant (*P* > 0.05); NR: not recorded; NE: not expected.

HPOO: high polyphenol olive oil.

MPOO: medium polyphenol olive oil.

LPOO: low polyphenol olive oil.

^*∗*^As suggested by Centre for Evidence-Based Medicine [29].

**Table 3 tab3:** Bergamot.

First author, year [ref]	Number of participants (M-F)	Age, y	Inclusion criteria	Dietary supplement		Duration (wk)	Mean or median baseline HDL-C (mmol/L)	Mean post-HDL-C (mmol/L)	Δ change (within groups) (*P* value)	MDΔ (between groups) (*P* value)	Study design (level of evidence)^*∗*^
				Intervention	Control						
Gliozzi, 2013 [45]	77 (NR-NR)	—	Mixed hypercholesterolemia, LDL-C levels of >4.14 mmol/L, and TG > 2.54 mmol/L	BPF: 1000 mg *Patients: 15*	Group A Rosuvastatin: 10 mg *Patients: 16* Group B Rosuvastatin: 20 mg *Patients: 16* Group C BPF: 1000 mg + Rosuvastatin: 10 mg *Patients: 15* Group D Placebo *Patients: 15*	4	NR	I: 1.16 ± 0.10 C: Group A 1.09 ± 0.08 Group B 1.24 ± 0.08 Group C 1.40 ± 0.10 Group D 0.98 ± 0.08	NR	I-C(A): NR (*P* < 0.05) I-C(B): NR (*P* < 0.05) I-C(C): NR (*P* < 0.05) I-C(D): NR (*P* < 0.05)	Prospective, open label, parallel-group, placebo-controlled study (Level 3)

Mollace, 2011 [46]	237 (NR-NR)	—	Group A (A1, A2, APLacebo) Hypercholesterolemia, HC cLDL levels ≥ 3.36 mmol/L Group B (B1, B2, BPLacebo) Hyperlipidemia (hypercholesterolemia and hypertriglyceridemia, HC/HT) Group C (C1, C2, CPLacebo) Hyperlipidemia and glycemia over 110 mg/dL, HC/HT/HG Group D or “poststatin.” Simvastatin therapy stopped due to muscular pain and a significant elevation of serum creatine-phosphokinase (CPK)	Group A1 BPF: 500 mg *Patients: 35* Group A2 BPF: 1000 mg *Patients: 37* Group B1 BPF: 500 mg *Patients: 14* Group B2 BPF: 1000 mg *Patients: 14* Group C1 BPF: 500 mg *Patients: 20* Group C2 BPF: 1000 mg *Patients: 19* Group D: BPF: 1500 mg *Patients: 32*	Placebo (APL + BPL + CPL) *Patients: 59*	4	NR	NR	NR	I(C1)-C: NR (*P* < 0.0001) I(C2)-C: NR (*P* < 0.0001) I(C2)-I(C1): NR (*P* < 0.05)	Randomized, double-blind, placebo-controlled study (Level 2)

MDΔ: mean difference Δ change.

I: intervention; C: control.

*P*: *P* value; NS: not significant (*P* > 0.05); NR: not recorded; NE: not expected.

^*∗*^As suggested by Centre for Evidence-Based Medicine [29].

**Table 4:** Artichoke.

First author, year [ref]	Number of participants (M-F)	Age (y)	Inclusion criteria	Dietary supplement		Duration (wk)	Mean or median baseline HDL-C (mmol/L)	Mean post-HDL-C (mmol/L)	Δ change (within groups) (*P* value)	MDΔ (between groups) (*P* value)	Study design (level of evidence)^*∗*^
				Intervention	Control						
Rondanelli, 2014 [47]	92 (41-51)	54.0 ± 7.8	Mild hypercholesterolaemia (5.4–7.0 mmol/L)	LE: 0.5 g/die *Patients: 46*	Placebo *Patients: 46*	8	I: 2.09 ± 0.55 C: 1.95 ± 0.64	NR	I: +0.207 (*P* < 0.05) C: +0.005 (*P* = NS)	+0.202 (*P* = 0.004)	Randomized, double-blind, placebo-controlled trial (Level 2)

Bundy, 2008 [48]	75 (27-48)	57.5 (SD NR)	Age over 50 years and BMI range of 20–25 kg/m^2^	LE: 1.28 g/die *Patients: 35*	Placebo *Patients: 38*	12	I: 1.57 ± 0.36 C: 1.53 ± 0.32	I: 1.56 ± 0.36 C: 1.54 ± 0.30	I: −0.01 (*P* = NS) C: +0.01 (*P* = NS)	−0.02 (*P* = NR)	Randomized, double-blind placebo-controlled trial (Level 2)

Nazni, 2006 [49]	30 (NR-NR)	40 (SD NR)	Diabetic type 2 with no insulin therapy	LE: 6 g/die *Patients: 15*	Placebo *Patients: 15*	12	I: 0.89 ± 0.08 C: 0.91 ± 0.08	I: 1.12 ± 0.08 C: 0.92 ± 0.08	I: 0.23 (*P* < 0.01) C: +0.01 (*P* = NS)	0.22 (*P* = NR)	Placebo-controlled trial (Level 3)

Rondanelli, 2013 [50]	55 (25-30)	54.1 ± 9.8	BMI from 25 to 35 kg/m^2^ with IFG (6.1–7.0 mmol/L), glycosylated haemoglobin < 7.0%, and no history of CVD	FBE: 0.6 g/die *Patients: 26*	Placebo *Patients: 29*	8	I: 1.38 ± 0.43 C: 1.53 ± 0.73	NR	I: +0.00 (*P* = NS) C: −0.04 (*P* = NS)	0.04 (*P* = NS)	Randomized, double-blind, placebo-controlled trial (Level 2)

Englisch, 2000 [51]	143 (47-96)	51.9 (SD NR)	Age between 18 and 70 years with TC > 7.3 mmol/L	DE: 1.8 g/die *Patients: 72*	Placebo *Patients: 71*	6	I: 1.17 ± 0.75 C: 1.20 ± 0.82	I: 1.13 ± 0.46 C: 1.21 ± 0.48	I: −0.04 (*P* = NR) C: +0.01 (*P* = NR)	−0.05 (*P* = NS)	Randomized, double-blind, placebo-controlled, multicentre clinical trial (Level 2)

MDΔ: mean difference Δ change.

I: intervention; C: control.

*P*: *P* value; NS: not significant (*P* > 0.05); NR: not recorded; NE: not expected.

LE: leaf extract.

FBE: flowering bud leaf extract.

DE: dry extract.

^*∗*^As suggested by Centre for Evidence-Based Medicine [29].
